# Digital psychosocial follow-up for survivors of childhood critical illness: protocol for a systematic literature review

**DOI:** 10.1186/s13643-025-03028-2

**Published:** 2025-12-23

**Authors:** Marte Hoff Hagen, Letizia Jaccheri, Sofia Papavlasopoulou, Gunnar Hartvigsen, Thorstein Sæter

**Affiliations:** 1 Department of Computer Science, Norwegian University of Science and Technology (NTNU), Sem Sælands vei 9, Trondheim, 7034 Norway; 2 Department of Computer Science, University of Tromsø – The Arctic University of Norway (UiT), Tromsø, Norway; 3 Children’s Clinic, St. Olavs Hospital, Trondheim University Hospital, Trondheim, Norway

**Keywords:** Childhood critical illness, Long-term follow-up care, Psychosocial, Digitalization

## Abstract

**Background:**

Survivors of childhood critical illness face a higher risk of long-term psychological and social challenges. Digital solutions have the potential to mitigate these issues. Thus, the objective of this systematic literature review is to examine a range of existing digital solutions for psychosocial follow-up for children who have survived a critical illness. The research questions are structured using the Population, Intervention, Comparison, and Outcome (PICO) model.

**Methods:**

A five-stage iterative process for systematic literature review in software engineering guided by Kitchenham will be used. The reporting of the review will adhere to the Preferred Reporting Items for Systematic reviews and Meta-Analyses (PRISMA) guidelines. The abstract and indexing database Scopus will be employed using a search strategy based on the PICO model. The automatic search will be supplemented by backward citation searching. The data will be managed using the EndNote software, with records selected according to eligibility criteria based on the PICO and Study design framework. This framework will also guide the data extraction process. All included studies will be assessed for risk of bias using the Mixed Methods Appraisal Tool (MMAT) and synthesized using Braun and Clarke’s six-phase thematic analysis framework for qualitative data and meta-analysis for quantitative data when possible. For the meta-analysis, the effect measures will be calculated using forest plots based on the DerSimonian and Laird method. Heterogeneity will be explored through meta-regression, and the robustness of the meta-analysis results will be assessed using sensitivity analysis. The risk of bias due to missing results in the synthesis will be assessed using funnel plots. The Grading of Recommendations Assessment, Development, and Evaluation (GRADE) approach will be used to evaluate the certainty of the evidence provided by the review.

**Discussion:**

This review will provide insights into the existing digital psychosocial follow-up solutions for survivors of childhood critical illness, potentially leading to enhanced solutions and improved quality of life for this group.

**Systematic review registration:**

PROSPERO CRD42022364703

**Supplementary Information:**

The online version contains supplementary material available at 10.1186/s13643-025-03028-2.

## Background

A *childhood critical illness* could be defined as any disease requiring treatment at a pediatric intensive care unit [1, 2]. Survivors of childhood critical illness have an increased risk of long-term psychosocial issues because intensive treatment procedures and hospitalization in childhood can affect a child psychologically, socially, cognitively, and physically [3–5]. Therefore, there exist “psychological and social services and interventions that enable patients, their families, and health care providers to optimize biomedical health care and to manage the psychological/behavioral and social aspects of illness and its consequences [...] to promote better health” ([6], p. 9), called *psychosocial follow-up*.

Digital solutions for such psychosocial follow-up for survivors of childhood critical are increasing [7, 8]. Technology to provide psychosocial support for children and young people with long-term conditions has proceeded parallel to technological advances, quickly adopted by health professionals [7]. Game technologies for pediatric patients used for distraction, motivation, socialization, education, and emotional coping have expanded since 2010 [8]. The literature indicates that these technologies could improve psychosocial aspects for youths [7, 9–11]—despite limited available research [11].

While relevant previous systematic literature reviews (SLRs) have targeted specific technology types (e.g., video games [8] and virtual reality [12]), this SLR aims to investigate all kinds of digital psychosocial follow-up solutions (e.g., software, robot, and telehealth). This SLR also goes beyond other recent SLRs that have examined digital psychosocial follow-up solutions for chronic conditions [7, 10, 11]. According to the definition of childhood critical illness, one could be a survivor of a childhood critical illness without having a chronic disease. Thus, the main objective of this research is to *explore the existing literature on the range of digital solutions for psychosocial follow-up for children who survived a critical illness*. An SLR is a suitable method to identify and summarize the available relevant research [13]. The objective will be investigated by the research questions (RQs) presented in Table 1. These RQs were formulated based on the Population, Intervention, Comparison, and Outcome (PICO) framework [14], a well-used tool to frame clinical RQs in SLRs [12, 13, 15–17].

**Table 1:** Overview of the research questions (RQs) based on the Population, Intervention, Comparison, and Outcome framework

Element	RQ1: Which existing digital solutions currently help survivors of childhood critical illness psychosocially?
Population	**RQ1.1:** What are the target groups for these solutions?
Intervention	**RQ1.2:** How are these solutions designed?
Comparison	**RQ1.3:** How are these solutions evaluated?
Outcome	**RQ1.4:** How effective are these solutions?

## Methods

Due to the digital focus of this SLR, the original guidelines for software engineering [13] will be followed. Relevant principles from the Cochrane Handbook for Systematic Reviews of Interventions [18] will also be consulted to ensure alignment with best practices, particularly regarding risk of bias assessment and evidence grading. The Preferred Reporting Items for Systematic reviews and Meta-Analyses (PRISMA) Statement’s standards [19] will guide the reporting of the SLR. For reporting this protocol, the PRISMA-Protocol Statement’s checklist has been followed (see Additional file 1). This protocol was documented on 04.10.2022 in the International Prospective Register of Systematic Reviews with registration number CRD42022364703 [20]. If protocol amendments are needed, a table with the date, the description, and the rationale of each change will be created. Changes will not be incorporated into the protocol but described in the final SLR.

### Identification of research

An automatic search will be conducted in the database Scopus^1^, the broadest covered interdisciplinary abstract and citation database [21], including scholarly literature in the various relevant disciplines of computer science (physical science), medicine (health science), and psychology (social science) [22]. This database includes MEDLINE-indexed journals, the core component of PubMed [23].

The search string (Table 2) was developed based on the PICO model, which is also frequently used to develop search strings [15]. This PICO model combined different synonyms of the keywords to optimize the search string and include more relevant studies in the review. The synonyms were found by investigating similar SLRs identified in pilot searches. An overview of the development of the search string is included in Additional file 2. The search string was fulfilled by adding the logical operator AND between each group of synonyms for a keyword (i.e., row in Table 2). The synonyms of the keyword *critical illness survivors* encompassed synonyms for *chronic illness*. To avoid scope narrowing, the primary strategy will not include disease-specific terms except for synonyms for the target group of this SLR’s associated PhD project [24]: *survivors of Hirschsprung’s disease or Anorectal Malformation*.

This latter list of synonyms is complete and found below the dashed line in Table 2, representing the logical operator OR.

**Table 2:** Overview of the Population, Intervention, Comparison, Outcome, and Study design model used to form the search string

Element	Keyword	Synonyms
Population	Critical illness survivors	{critical illness} OR {critical illnesses} OR {critical disease} OR {critical diseases} OR {critically ill} OR {chronic illness} OR {chronic illnesses} OR {chronic disease} OR {chronic diseases} OR {chronic condition} OR {chronic conditions} OR {chronic disorder} OR {chronic disorders} OR {chronic infection} OR {chronic infections} OR {noncommunicable disease} OR {noncommunicable diseases} OR {non-communicable disease} OR {non-communicable diseases} OR {noncommunicable condition} OR {noncommunicable conditions} OR {non-communicable condition} OR {non-communicable conditions} OR {long term condition} OR {long term conditions} OR {long-term condition} OR {long-term conditions} OR {long term disease} OR {long term diseases} OR {long-term disease} OR {long-term diseases} OR survivor* OR hospitalization
		- - - - - - - - - - - - - - - - - - - - - - - - - - - - - - - - - - - - - - - - - - - - - - - - - - - - -
		Hirschsprung* OR {colonic aganglionosis} OR {congenital megacolon} OR {aganglionic megacolon} OR {intestinal aganglionosis} OR {Anorectal Malformations} OR {Imperforate Anus} OR {Anal Atresia} OR {Anal Membrane} OR {Anal Stenosis} OR {Rectoperineal Fistula} OR {Ectopic Anus} OR {Perineal Anus} OR {Rectovestibular Fistula} OR {Rectoprostatic Fistula} OR {Rectobulbar Fistula} OR {Bladder Neck Fistula}
	Children	pediatric OR child* OR underage* OR boy* OR girl* OR infant* OR toddler* OR preteen* OR teen OR teens OR preadolescent* OR adolescent*
Intervention	Digital	digital OR online OR software OR app OR apps OR application* OR mhealth OR m-health OR e-health OR ehealth OR telehealth OR telemedicine OR telecounsel* OR teletherapy* OR technolog* OR robot* OR {internet of things} OR {machine learning} OR {artificial intelligence} OR {augmented reality} OR {virtual reality} OR {health information system} OR web* OR smartphone* OR smartwatch* OR tablet*
Comparison		
Outcome	Psychosocial follow-up	psychosocial OR psychological OR social OR emotional OR spiritual OR mental OR well-being OR {quality of life}

To mitigate the risk of missing relevant studies not indexed in Scopus, the automatic search will be supplemented with backward citation searching by manually hand-searching reference lists of the relevant studies identified in the automatic search to identify additional studies of interest. This strategy aims to reduce potential systematic bias associated with using a single database.

The bibliographic management software EndNote 20.4^2^ will be utilized to handle the high number of records and remove duplicates identified by the search.

### Study selection

The study inclusion and exclusion criteria were defined by piloting the study selection process and the Population, Intervention, Comparison, Outcome, and Study design (PICOS) framework, presented in Table 3. This framework is the aforementioned PICO framework with an addition of a study design dimension, an appropriate dimension for study selection and extraction [18, 25, 26]. These study design criteria will mainly be excluded automatically in Scopus and EndNote.

**Table 3:** Overview of the study inclusion and exclusion criteria, based on the Population, Intervention, Comparison, Outcome, and Study design framework

Criteria	Inclusion	Exclusion	RQ
Population	The main study population is survivors of a disease requiring treatment at a pediatric intensive care unit	Adult participants (age$\ge$18 years old)	RQ1.1
		No study population	
Intervention	The main study intervention is a digital solution	No study intervention	RQ1.2
Comparison	Any		RQ1.3
Outcome	Psychosocial	No study outcome	RQ1.4
	Mental health		
	Quality of life		
	Psychological		
	Well-being		
	Emotional		
	Spiritual		
	Social		
Study Design	Published after 2009	Papers in press	
	English language	Duplicated studies	
		No full-text available	
		Protocols	
		Reviews	
		Editorials	
		Letters notes	
		PhD theses	
		Books	
		Book chapters	
		Reports	
		Short surveys	
		Conference proceedings	
		Conference papers	

*RQ *research question

This research defines children as Article 1 in the United Nations Convention on the Rights of the Child: “every human being below the age of eighteen years” [27]. 2010 was chosen as a lower time limit since it represents a paradigm shift in app development as the start of significant app growth because Apple released its Software Development Kit in 2009 [28].

One reviewer will narrow down the relevant studies by categorizing them with the grouping function in EndNote, following the eligibility criteria. This study selection process will be done in two iterations of excluding: one based on abstract and title only and one on full text. To ensure methodological rigor, regular consensus meetings with the review team will be held throughout the study selection process.

### Data extraction

The data collection, which involves extracting data from primary studies, will be carried out using the qualitative data analysis software NVivo Release 1.7^3^ and the spreadsheet software Microsoft Excel version 16.59^4^. This data extraction is based on the PICOS framework, as presented in Table 4. One reviewer will do the extraction independently but regularly discuss it with the other reviewers to reach consensus in the review team and reduce bias. In addition, a random subset of studies will be cross-checked by one of the other reviewers to validate consistency. Any discrepancies will be resolved through discussion among the reviewers. The main outcomes of the SLR will be *the psychosocial effects of the intervention*. Based on research regarding measuring psychosocial health for children [29–32], the included studies are expected to report the effect both quantitatively (e.g., questionnaires and observations) and qualitatively (e.g., interviews and open-ended questionnaires). Eventual additional outcomes will be reported under the item *Other findings*.

**Table 4:** Overview of the data extraction form, based on the Population, Intervention, Comparison, Outcome, and Study design framework

Focus area	Data	RQ
Population	Sample size	RQ1.1
	Medical conditions	
	Age distribution	
	Sex distribution	
	Ethnic distribution	
	Geographical distribution	
	Social demography	
	Inclusion criteria in the study	
Invention	Type	RQ1.2
	Context	
	Interaction mechanism	
	P5 medicine items	
Comparison	Research objective	RQ1.3
	Research questions	
	Research strategy	
	Data gathering method	
	Data analysis	
	Evaluation factors	
	Duration	
	Limitations	
	Risk of bias assessment	
Outcome	Measurement type	RQ1.4
	Psychosocial needs	
	Psychosocial theories	
	Psychosocial effect	
	Other findings	
	Implications	
	Data	
Study Design	Publication channel	
	Publication year	

*RQ* research question

The item *P5 medicine items* will extract if the intervention in the study is *preventive*, *personalized*, *predictive*, *participative*, and/or *psycho-cognitive*, the five items in medical informatics’ theoretical framework of P5 medicine [33].

### Study quality assessment

Because of the expected variance in the study design used in the primary studies, they will be assessed for quality in the form of risk of bias by the Mixed Methods Appraisal Tool (MMAT) version 2018 [34], recently used in a relevant SLR [35]. This tool is validated [36] and provides customized questions set on study level to judge each study type within its methodological domain by addressing the appropriateness of the aim of the study, the data collection, the data analysis, and the presentation of the findings [34]. One reviewer will independently apply the tool to each study and discuss the judgments regularly with the other reviewers to achieve an agreement between the reviewers.

### Data synthesis

The outcome type identified in the *Data Extraction* section and the study design identified in the *Study Quality Assessment* section determined how a study will be synthesized.

The qualitative data will be analyzed through Braun and Clarke’s six-phase framework of thematic analysis, which extracts data and then systematically codes, organizes, and interprets it [37]. An inductive approach will be utilized to ensure the generated themes are linked to the data [37]. One reviewer will do this synthesis but regularly discuss it with the other reviewers to ensure consensus within the review team.

The quantitative data will be analyzed independently by one reviewer using meta-analysis. The meta-analyses will be performed only when possible (i.e., when two or more studies are sufficiently homogeneous regarding study design and outcomes and have appropriate quantitative data available for calculation). Raw data will be used when available, and study authors will be contacted by mail to request missing data necessary for the meta-analysis. The request will be deemed rejected if the mail is not answered within two weeks. When meta-analysis is impossible, the results will be reported qualitatively, as described above.

The statistics will be conducted on the spreadsheet software Microsoft Excel version 16.59 and the reproducible statistical software Stata version 17.0^5^ when necessary. For each group of studies with the same study design (e.g., cluster randomized trials, case-control, cohort studies), forest plots will be created to calculate the effect measures, including each study’s effect estimates and confidence intervals, the heterogeneity, and the summary estimate. The standardized mean difference will be used for continuous outcome measures since the studies used different scales. In contrast, a successful outcome’s risk ratio will be calculated for binary outcome measures. All effect measures will have 95% confidence intervals. The I-squared statistic will be utilized to compute the statistical heterogeneity level in the form of inconsistency. Pooled effect estimates are not calculated when heterogeneity is high (i.e., *I*$^{2}\ge 75$%). The inverse-variance method in the form of the random-effects statistic model DerSimonian and Laird [38] will be used because the heterogeneity between the studies is anticipated considerably due to the high variation of participants, interventions, and study designs.

Cases of heterogeneity among the results across the studies will be investigated with meta-regression of different sub-groups of factors that could affect the effect measure of the intervention (types of interventions, the participant’s medical conditions, and the participant’s gender) when possible (i.e., two or more appropriate data sets are available for calculation). The analyses will be conducted within-study contrasts so the data from one study can be included in more than one subgroup. Subgroup effects will be compared using the Wald-Type Test, where the null hypothesis is rejected for $p < .05$. Moreover, sensitivity meta-analyses will be conducted to assess robustness by calculating a successful outcome’s odds ratio for binary outcome measures. Lastly, funnel plots for meta-analyses will be generated to assess the risk of bias due to missing results in synthesis. If asymmetry is detected in the funnel plot, the studies’ characteristics will be reviewed to evaluate whether the asymmetry is caused by publication bias or other factors like heterogeneity of the studies.

The Grading of Recommendations Assessment, Development, and Evaluation (GRADE) approach will be utilized to assess the certainty of the evidence for studies contributing data to the meta-analyses [39]. This certainty of the evidence will be evaluated through the five domains (risk of bias, imprecision, inconsistency, indirectness, and publication bias) to be either very low, low, moderate, or high [39]. The GRADE pro GDT software^6^ will be used to visualize the assessment. All decisions to up- or down-grade the studies’ certainty will be communicated with comments and footnotes [39]. One reviewer will do the assessment and discuss the judgments regularly with the other reviewers to reach consensus.

## Discussion

The results of this review could be used to inform researchers, software practitioners, and clinicians regarding existing digital solutions for psychosocial follow-up for children who survived a critical illness. This knowledge can lead to better digital solutions and improved psychosocial follow-up and life quality for survivors of childhood critical illness.

A possible limitation of the SLR is that the meta-analysis could be limited by insufficient data or considerable heterogeneity in the populations, interventions, outcomes, and study design of the included studies with quantitative data. This may restrict the feasibility of conducting a meta-analysis. However, in such cases, qualitative data synthesis of these studies will be performed.

Despite efforts to improve methodological rigor by following comprehensive approaches aligned with the original guidelines for software engineering [13], the PRISMA Statement standards [19], and the Cochrane Handbook for Systematic Reviews of Interventions [18], several limitations need to be acknowledged. These will be transparently reported in the final review. First, some relevant studies might be missed. Because this review is part of a PhD project with strict time and resource constraints, the search does not fully adhere to Cochrane’s guidelines [18], as it is limited to a single database. This could result in missing relevant studies indexed in other databases such as PsycINFO^7^, CINAHL^8^, Embase^9^, CENTRAL^10^, and Web of Science^11^. However, Scopus offers broad coverage of relevant disciplines and includes MEDLINE-indexed journals. Furthermore, backward citation searching will be performed to address this limitation by identifying additional studies to improve comprehensiveness.

Moreover, one reviewer will perform the initial screening and data extraction instead of dual independent reviewing with interrater reliability reported, as recommended by Kitchenham [13] and Cochrane [18]. To minimize human error and subjective judgment, regular consensus meetings with the review team will be held. For data extraction, a random subset of studies will also be independently verified by a second reviewer to ensure consistency. Any disagreements will be resolved through discussion.

These limitations emphasize the need for future health research with more resources dedicated to ensuring methodological rigor by following best practices outlined in the Cochrane Handbook for Systematic Reviews of Interventions [18].

## Supplementary information

Additional file 1. PRISMA-P 2015 Checklist. PRISMA-P (Preferred Reporting Items for Systematic review and Meta-Analysis Protocols) 2015 Checklist.

Additional file 2. Development of Search Strategy. Overview of the development of the search string, based on the Population Intervention, Comparison, and Outcome model.

## Data Availability

Not applicable, as no data have been created for this protocol.

## Abbreviations

GRADE: Grading of Recommendations Assessment, Development, and Evaluation
PICO: Population, Intervention, Comparison, and Outcome
PICOS: Population, Intervention, Comparison, Outcome, and Study design
PRISMA: Preferred Reporting Items for Systematic reviews and Meta-Analyses
RQ: Research Question
SLR: Systematic Literature Review

## Footnotes

1 https://www.scopus.com

2 https://endnote.com

3 https://www.alfasoft.com/en/products/statistics-and-analysis/nvivo.html

4 https://www.microsoft.com/en-ww/microsoft-365/excel

5 https://www.statanordic.com

6 https://gdt.gradepro.org

7 https://www.apa.org/pubs/databases/psycinfo

8 https://about.ebsco.com/products/research-databases/cinahl-database

9 https://www.elsevier.com/products/embase

10 https://www.cochranelibrary.com/central/about-central

11 https://clarivate.com/academia-government/scientific-and-academic-research/research-discovery-and-referencing/web-of-science/
