# Supplementary material for: Digital psychosocial follow-up for survivors of childhood critical illness: protocol for a systematic literature review
Source: Syst Rev. 2025 Dec 23;15:30. doi: 10.1186/s13643-025-03028-2 (PMC12838011; doi:10.1186/s13643-025-03028-2)
Supplement: Supplementary file 2 — Additional file 2. Development of Search Strategy. Overview of the development of the search string, based on the Population Intervention, Comparison, and Outcome model. [file 13643_2025_3028_MOESM2_ESM.pdf]

	Population		Intervention	Comparison	Outcome	Comment
#1	"critical illness" OR "critical disease" OR "critically ill" OR "chronic illness" OR "chronic disease" OR "chronic condition" OR "chronic disorder" OR "chronic infection"	pediatric	digital OR software OR app OR application OR mhealth OR m-health OR technolog* OR ICT			
#2		pediatric OR child*				Include the term *child**
#3	"critical illness" OR "critical disease" OR "critically ill" OR "chronic illness" OR "chronic disease" OR "chronic condition" OR "chronic disorder" OR "chronic infection" OR hospitalization					Include the term *hospitalization*
#4	"critical illness" OR "critical disease" OR "critically ill" OR "chronic illness" OR "chronic disease" OR "chronic condition" OR "chronic disorder" OR "chronic infection"				psychosocial AND rehabilitation	Include terms for outcome
#5					psychosocial OR rehabilitation	
#6					psychosocial OR "follow-up"	
#7					"psychosocial follow-up"	
#8	"critical illness" OR "critical disease" OR "critically ill" OR "chronic illness" OR "chronic disease" OR "chronic condition" OR "chronic disorder" OR "chronic infection" OR "noncommunicable disease" OR "noncommunicable condition" OR "long term condition" OR "long term disease" OR survivor* OR hospitalization	pediatric OR child* OR underage* OR boy* OR girl*	digital OR online OR software OR app OR apps OR application* OR mhealth OR m-health OR e-health OR ehealth OR telehealth OR telemedicine OR telecounsel* OR teletherapy* OR technolog* OR ICT OR robot* OR IOT OR "internet of things" OR "machine learning" OR ML OR AI OR "artificial intelligence" OR "augmented reality" OR AR OR "virtual reality" OR VR OR "health information system" OR device* OR web* OR mobile OR smartphone* OR smartwatch* OR tablet*		psychosocial OR psychological OR social OR emotional OR spiritual OR mental OR well-being OR QOL OR "quality of life"	Include more synonyms
#9	"critical illness" OR "critical disease" OR "critically ill" OR "chronic illness" OR "chronic disease" OR "chronic condition" OR "chronic disorder" OR "chronic infection" OR "noncommunicable disease" OR "noncommunicable condition" OR survivor* OR "long term condition" OR "long term disease" OR hospitalization OR "Hirschsprung Disease" OR "Hirschsprung's disease" OR "Hirschsprung-associated enterocolitis" OR HAEC OR HSCR OR "colonic aganglionosis" OR "congenital megacolon" OR "aganglionic megacolon" OR "intestinal aganglionosis" OR "Anorectal Malformations" OR "Imperforate Anus" OR "Anal Atresia" OR "Anal Membrane" OR "Anal Stenosis" OR "Rectoperineal Fistula" OR "Ectopic Anus" OR "Perineal Anus"					Include target group
#10	"critical illness" OR "critical illnesses" OR "critical disease" OR "critical diseases" OR "critically ill" OR "chronic illness" OR "chronic illnesses" OR "chronic disease" OR "chronic diseases" OR "chronic condition" OR "chronic conditions" OR "chronic disorder" OR "chronic disorders" OR "chronic infection" OR "chronic infections" OR "noncommunicable disease" OR "noncommunicable diseases" OR "non-communicable disease" OR "non-communicable diseases" OR "noncommunicable condition" OR "noncommunicable conditions" OR "non-communicable condition" OR "non-communicable conditions" OR "long-term condition" OR "long-term conditions" OR "long-term condition" OR "long-term conditions" OR "long term disease" OR "long term diseases" OR "long-term disease" OR "long-term diseases" OR survivor* OR hospitalization OR Hirschsprung* OR "colonic aganglionosis" OR "congenital megacolon" OR "aganglionic megacolon" OR "intestinal aganglionosis" OR "Anorectal Malformations" OR "Imperforate Anus" OR "Anal Atresia" OR "Anal Membrane" OR "Anal Stenosis" OR "Rectoperineal Fistula" OR "Ectopic Anus" OR "Perineal Anus"	pediatric OR child* OR underage* OR boy* OR girl* OR infant* OR toddler* OR preteen* OR teen OR teens OR preadolescent* OR adolescent*				Include more synonyms for population
#11			digital OR online OR software OR app OR apps OR application* OR mhealth OR m-health OR e-health OR ehealth OR telehealth OR telemedicine OR telecounsel* OR teletherapy* OR technolog* OR robot* OR "internet of things" OR "machine learning" OR "artificial intelligence" OR "augmented reality" OR "virtual reality" OR "health information system" OR web* OR smartphone* OR smartwatch* OR tablet*		psychosocial OR psychological OR social OR emotional OR spiritual OR mental OR well-being OR"quality of life"	Remove abbreviations and other words contributing to a high number of unrelevant hits
#12	{critical illness} OR {critical illnesses} OR critical disease OR critical diseases OR {critically ill} OR {chronic illness} OR {chronic illnesses} OR {chronic disease} OR {chronic diseases} OR {chronic condition} OR {chronic conditions} OR {chronic disorder} OR {chronic disorders} OR {chronic infection} OR {chronic infections} OR {noncommunicable disease} OR {noncommunicable diseases} OR {non-communicable disease} OR {non-communicable diseases} OR {noncommunicable condition} OR {noncommunicable conditions} OR {non-communicable condition} OR {non-communicable conditions} OR survivor* OR {long-term condition} OR {long-term conditions} OR {long-term condition} OR {long-term conditions} OR {long term disease} OR {long term diseases} OR {long-term disease} OR {long-term diseases} OR hospitalization OR Hirschsprung* OR {colonic aganglionosis} OR {congenital megacolon} OR {aganglionic megacolon} OR {intestinal aganglionosis} OR {Anorectal Malformations} OR {Imperforate Anus} OR {Anal Atresia} OR {Anal Membrane} OR {Anal Stenosis} OR {Rectoperineal Fistula} OR {Ectopic Anus} OR {Perineal Anus} OR {Rectovestibular Fistula} OR {Rectoprostatic Fistula} OR {Rectobulbar Fistula} OR {Bladder Neck Fistula}		digital OR online OR software OR app OR apps OR application* OR mhealth OR m-health OR e-health OR ehealth OR telehealth OR telemedicine OR telecounsel* OR teletherapy*OR technolog* OR robot* OR {internet of things} OR {machine learning} OR {artificial intelligence} OR {augmented reality} OR {virtual reality} OR {health information system} OR web* OR smartphone* OR smartwatch* OR tablet*		psychosocial OR psychological OR social OR emotional OR spiritual OR mental OR well-being OR {quality of life}	Include more synonyms for target group and adjust the search string to Scopus by changing quotation mark to braces
